# Immunopathogenesis and Cellular Interactions in Human T-Cell Leukemia Virus Type 1 Associated Myelopathy/Tropical Spastic Paraparesis

**DOI:** 10.3389/fmicb.2020.614940

**Published:** 2020-12-22

**Authors:** Sepehr Aghajanian, Majid Teymoori-Rad, Ghazale Molaverdi, Sayed-Hamidreza Mozhgani

**Affiliations:** ^1^Student Research Committee, Alborz University of Medical Sciences, Karaj, Iran; ^2^Department of Virology, School of Public Health, Tehran University of Medical Sciences, Tehran, Iran; ^3^Non-communicable Diseases Research Center, Alborz University of Medical Sciences, Karaj, Iran; ^4^Department of Microbiology, School of Medicine, Alborz University of Medical Sciences, Karaj, Iran

**Keywords:** HAM/TSP, HTLV-1-associated myelopathy, pathogenesis, immunology, human T-lymphotropic virus

## Abstract

HTLV-1-Associated Myelopathy/Tropical Spastic Paraparesis (HAM/TSP) is a neuropathological disorder in 1–3% of individuals infected with Human T-lymphotropic virus 1 (HTLV-1). This condition is characterized by progressive spastic lower limb weakness and paralysis, lower back pain, bladder incontinence, and mild sensory disturbances resembling spinal forms of multiple sclerosis. This disease also causes chronic disability and is therefore associated with high health burden in areas where HTLV-1 infection is endemic. Despite various efforts in understanding the virus and discovery of novel diagnostic markers, and cellular and viral interactions, HAM/TSP management is still unsatisfactory and mainly focused on symptomatic alleviation, and it hasn’t been explained why only a minority of the virus carriers develop HAM/TSP. This comprehensive review focuses on host and viral factors in association with immunopathology of the disease in hope of providing new insights for drug therapies or other forms of intervention.

## Introduction

Human T-lymphotropic virus type 1 (HTLV-1), discovered in 1980, is a retrovirus from the Orthoretrovirinae subfamily of Deltaretrovirus genus. HTLV-1 has seven reported subtypes (subtypes a to g), identified by their related geographic regions and genetic variability (Verdonck et al., 2007; Bangham, 2018; Nozuma and Jacobson, 2019). The virus primarily infects CD4+ T-cells and causes lifelong infections in humans. It propagates mostly, during the chronic phase of infection, through clonal expansion of infected cells and not by *de novo* infection with cell-free virions, a unique trait even among other genera of retroviruses. The main routes of transmission are through sexual intercourse, breastfeeding, cellular blood products, and to a lesser extent, organ transplantations and perinatal infections (Kawase et al., 1992; Armstrong et al., 2012; Bangham et al., 2015; Rosadas and Taylor, 2019). While most of the infected subjects maintain normal life and are asymptomatic carriers (AC), the virus disrupts the immune system function in some individuals, leading to the development of malignant neoplasms such as Adult T-cell leukemia/leukemia (ATLL) and is associated with immune hypersensitivity conditions like uveitis, arthritis, atherosclerosis, Sjogren’s syndrome, thyroiditis, polymyositis, infective dermatitis, and most notably HTLV-1 associated myelopathy/Tropical spastic paraparesis (HAM/TSP) (Abolbashari et al., 2018; Caswell and Manavi, 2020; Schierhout et al., 2020; Umekita and Okayama, 2020).

HAM/TSP is described as a chronic paraparesis with an insidious onset and a similar clinical picture to Multiple sclerosis (Araujo and Silva, 2006). The disease is characterized by progressive demyelination and neuronal loss in CNS. Lesions occur mainly in posterior, and lateral columns of middle to lower thoracic parts of spinal cord (Iwasaki, 1990; Osame, 2002; Bangham et al., 2015). Spinal cord atrophy and perivascular and parenchymal infiltration of mononuclear cells in CNS have also been observed and are regarded as pathological hallmarks of HAM/TSP (Osame, 2002; Bangham et al., 2015; Nozuma and Jacobson, 2019). Patients present with lower limb paraparesis, lower back pain, dysautonomia, mild sensory disturbances, constipation and cognitive decline (Araujo and Silva, 2006; Araujo and Wedemann, 2019; Tsutsumi et al., 2019). Treatment is primarily focused on control of pain, muscle spasms, and symptomatic treatment of autonomic disturbances, namely, bladder incontinence, constipation, and erectile dysfunction (Bangham et al., 2015). HAM/TSP has a shorter latency than ATLL, ranging from months to decades before the development of symptoms (Olière et al., 2011).

Since the discovery of HTLV-1 as the etiologic agent of HAM/TSP (Poiesz et al., 1980) and establishment of diagnostic criteria for the disease by World Health Organization (World Health Organization. Regional Office for the Western Pacific, 1988), innovative advances in mapping the viral interactions with the host have led to the discovery of new viral mechanisms in the pathogenesis of HTLV-1 diseases and novel insights in diagnosing the disease via pathological markers, but still, the mechanisms underlying tissue damage and disease development are not fully understood. This review characterizes the pathological mechanisms in developing HAM/TSP with emphasis on HTLV-1 genes, structure, and cell-cell and virus-cell interactions throughout the host.

## Host Factors in Development of HAM/TSP

Among the host-dependent factors, genotype of major histocompatibility complex/human leukocyte antigen 1 (MHC-I/HLA class I) is one of the factors determining the effectiveness of cell-mediated responses and viral burden which is described as the number of viral copies integrated into the host genome in peripheral blood mononuclear cells (Proviral load; PVL) in individuals. Class-I heterozygosity may also reduce PVL in infected subjects (Jeffery et al., 2000). Certain class II HLAs are also associated with HAM/TSP by increasing CD4+ T-cell activity and subsequent tissue damage (Jeffery et al., 1999; Bangham et al., 2015). Comparatively, protective HLAs are more likely to be detected in ACs and predisposing HLAs are more frequent in patients with HAM/TSP. For example, the protective effects of HLA-A^∗^02:07 and HLA-C^∗^08 and the detrimental effects of HLA-B^∗^54 has been demonstrated in southern Japan (Boelen et al., 2018). HLA-Cw^∗^08 was similarly seen in both ACs and HAM / TSP patients in a Brazilian study, but was associated with a protective effect in Japan and higher susceptibility to HAM/TSP in Iran (Vallinoto et al., 2019). Class II HLA-DRB1^∗^0101 has been identified as a predisposing factor to the disease in Japan, Iran, and Spain and it has been shown that the effect is exacerbated in the absence of the protective effect of HLA-A^∗^02 (Jeffery et al., 1999; Saito, 2019; Vallinoto et al., 2019). Haplotype HLA-B^∗^0702-Cw^∗^0702-DRB1^∗^0101-DQB1^∗^0501 is also predisposing in the absence of HLA-A^∗^02 (Catalan-Soares et al., 2009). In addition, the protective effects of HLA-B^∗^07 in Spain and HLA-A^∗^0201 and HLA-Cw^∗^0801 in Japan are documented as well (Vallinoto et al., 2019). The predisposing effects HLA-B^∗^5401 have been proven, but are not observed in Brazil (Catalan-Soares et al., 2009; Bangham et al., 2015; Enose-Akahata and Jacobson, 2019).

The effect of class 1 HLAs on disease progression in HTLV-1 infection is affected by Inhibitory killer cell immunoglobulin-like receptors (iKIRs), which are expressed on natural killer (NK) cells as well as CD8+ T-cells. iKIR enhance the HLA protective and detrimental effects through binding with their HLA class 1 ligands and increasing the lifespan of CD8+ T-cells (Boelen et al., 2018). The abundance of HLAs involved in determining the course of infection and disease progression indicates that the development of HAM/TSP is reliant on a dynamic interaction between the host and the virus. Polymorphisms and genetic variability in other genes also influence the development of HAM/TSP; some of which are listed in Table 1. It would be an interesting topic to test whether individuals bearing more genetic risk factors would benefit from a more aggressive treatment plan.

**TABLE 1:** Predisposing and protective factors for development of HAM/TSP.

**Predisposing and protective factors for development of HAM/TSP**	
**Predisposing factors**	**Protective factors**
Longer CA repeat alleles of MMP-9 promoter (Saito and Bangham, 2012)	SDF-1 promoter +801 A allele (Saito and Bangham, 2012)
TNF-α promoter −963 A allele (Saito and Bangham, 2012)	IL-10 promoter −592 A allele (Saito and Bangham, 2012)
IL-6 promoter −634 G/C allele (Saito and Bangham, 2012; Vallinoto et al., 2019)	IL-15 +191 C allele (Saito and Bangham, 2012)
IL-28B single nucleotide polymorphism (SNP) rs12979860 (Assone et al., 2014)	Vitamin D receptor gene SNP ApaI AA genotype (Saito et al., 2005)
LT-α* A/G (Vallinoto et al., 2019)	Killer Immunoglobulin-like Receptors (KIR) 2DL2 (Vallinoto et al., 2019)
TNFR2* 196R (Vallinoto et al., 2019)	IL18 _607*CC (Vallinoto et al., 2019)
HLA-B*54 (Boelen et al., 2018)	HLA-A*02:07 (Boelen et al., 2018)
HLA-DRB1*0101 (Jeffery et al., 1999)	HLA-C*08 (Boelen et al., 2018)
HLA-Cw*08 (Vallinoto et al., 2019)	HLA-Cw*08 (Vallinoto et al., 2019)
HLA-B*0702-Cw*0702-DRB1*0101-DQB1*0501 Haplotype without the presence of (HLA-A*02) (Catalan-Soares et al., 2009)	HLA-B*07 (Vallinoto et al., 2019)
HLA-B*5401 (Catalan-Soares et al., 2009)	HLA-A*0201 (Vallinoto et al., 2019)
	HLA-Cw*0801 (Vallinoto et al., 2019)

*Adapted from Saito and Bangham (2012), Immunopathogenesis of human T-cell leukemia virus type-1-associated myelopathy/tropical spastic paraparesis: recent perspectives.*

Host factors that may precipitate ACs to HAM/TSP may not be limited to genetic factors. Although no concrete study on environmental factors for HAM/TSP exists, it is believed that HAM/TSP is more likely than ATLL to develop as the result infection via blood transfusion, whereas ATLL develops more often in recipients of vertical transmission (Oger, 2007). This observation could be the result of the greater latency period of ATLL (Olière et al., 2011), which would manifest as higher incidence of the disease in individuals infected in the earlier stages of life (Vertical transmission).

## HTLV-1 Virology, Gene Expression, and Associated Proteins

**FIGURE 1:**
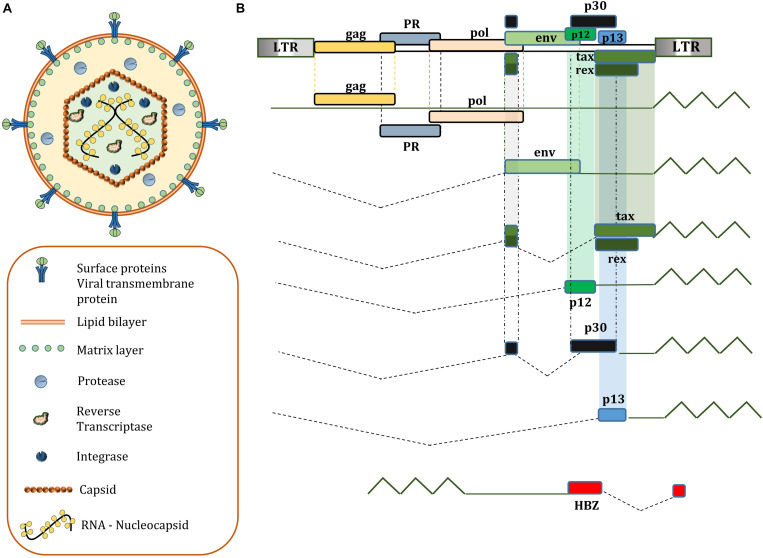
**(A)** Structure of a mature HTLV-1 virion; **(B)** genetic composition of HTLV-1; HBZ, p12, p13, p30 accessory proteins are not essential for immortalization of T lymphocytes *in vitro*, but have a significant role in viral latency and persistent infection in humans.

HTLV-1 enveloped virions contain the 9-kb positive-sense RNA which serves as a template for the provirus. The RNA is packaged in the viral core with the viral nucleocapsid protein, surrounded by capsid and matrix proteins. The virus relies on cell-to-cell contact of infected host cells to spread, which are usually CD4+ or CD8+ T-cells (Hoshino, 2012). The provirus encodes *gag*, *pro*, *pol*, and *env* genes observed in other retroviruses. Matrix, Capsid, and Nucleocapsid proteins are products of cleavage of *gag* encoded p55 by viral protease. The Reverse Transcriptase, RNase H, and Integrase is encoded by the *pol* gene. Moreover, HTLV-1 virion surface receptor units gp46 (Surface unit) and gp21 (transmembrane unit) are synthesized from cleaving gp61, the precursor protein encoded by the *env* gene; While HTLV-1 protease is encoded by the *pro* gene. In addition to these structural proteins, HTLV-1 genome also includes a pX region containing viral regulatory and accessory genes for following proteins with partially overlapping open reading frames: Tax, Rex, p8, p12, p13, p21, p30, and HTLV-1 Basic Zipper Factor (HBZ) (Journo et al., 2009; Giam and Semmes, 2016; Soltani et al., 2019; Figure 1). At each end of the genome is a long terminal repeat (LTR) sequence that regulates various stages in the cycle of infection. The upstream 5′LTR acts as a TATA-box-containing inducible promoter for viral transcription of plus-strand (Kulkarni and Bangham, 2018), whereas HBZ is translated from 3′LTR in minus-strand by a TATA-less, Sp-1 dependent promoter (Yoshida et al., 2008).

## Tax

Tax as a viral protein affects several cellular pathways, leading to changes in host gene expression, T-cell differentiation, and modification of immune response. The three tandem nucleotide repeats called tax responsive elements (TRE) in the 5′LTR promoter regulate plus-strand expression (Giebler et al., 1997) through binding of proteins of the ATF/CREB family. Plus-strand expression is enhanced by transcription of tax by increasing binding of CREB to TREs through a positive feedback loop, enabling transactivation and spontaneous viral replication (Kulkarni and Bangham, 2018). Furthermore, tax is responsible for modulation of various signaling pathways; including I kappa B kinase, nuclear factor-kappa B (NF-κB), AP-1, JAK1/STAT3, JAK3/STAT5, IRF3, IRF4, and TGF-β signaling (Grant et al., 2008; Olière et al., 2010; Suzuki et al., 2010; Kataoka et al., 2015; Tanaka and Matsuoka, 2018). Tax also acts as an oncoprotein by inhibiting tumor suppressors (p53, Bcl11B, and TP53INP1) and activating cyclin-dependent kinases, both of which lead to increased cell proliferation (Carpentier et al., 2015). Moreover, impaired antiviral signaling in HTLV-1 infections is partially because of Tax-mediated induction of Suppressor of cytokine signaling 1 and reduction of MHC-I in infected cells (Olière et al., 2011). Reduction of interferon type I production via IRF3 phosphorylation by Tax also impairs host viral defense against HTLV-1 (Olière et al., 2011; Bangham, 2018). Furthermore, Tax-mediated expression of proinflammatory mediators and apoptosis is strongly associated with the neuropathology of CNS in HAM/TSP (Banerjee et al., 2007b). Importance of Tax in generating the immune response is emphasized; as while CD4+ T-cells response is predominantly directed toward Env products, the immunodominant antigen recognized by CD8+ T-cells is the Tax protein (Bangham et al., 2015). The reader is referred to review articles for further details regarding Tax interaction with host pathways (Boxus et al., 2008; Matsuoka and Jeang, 2011; Fochi et al., 2018).

## HBZ

The functions of HBZ protein are usually in contrast to Tax. HBZ suppresses viral transcription via binding with CREB and selective inhibition of the NF-κB and AP-1 pathways via interacting with p65 and c-Jun, respectively (Tanaka and Matsuoka, 2018; Fochi et al., 2019). HBZ also transactivates the expression of TGF-β, whereas Tax has the opposite effect (Bangham, 2018). These opposing interactions are regarded as advantageous for the virus, because Tax induces a strong immune response in both CD4+ and CD8+ T-cells. The inhibition of transcription of viral structural genes increases viral latency and persistence in infected cells (Bangham et al., 2019). HBZ also downregulates apoptosis and autophagy by reducing FoxO3a-mediated transcription of Bim and mTOR inhibitor, respectively. These regulatory functions contribute to cell proliferation, maintenance of viral reservoirs, and cancer development in ATLL (Mukai and Ohshima, 2014; Vescovo et al., 2020). Although both Tax and HBZ are transcribed in bursts in a single clone (Billman et al., 2017); HBZ is expressed in most of the infected cells (Satou et al., 2006), whereas Tax expression is silenced in various clones through epigenetic and genetic modifications and other viral regulatory genes such as *rex*, *p30*, and *hbz* (Nicot et al., 2004; Takeda et al., 2004; Taniguchi et al., 2005).

Interestingly, only a small fraction of HBZ mRNA is translated into HBZ protein in PBMCs of HAM/TSP subjects (Shiohama et al., 2016). Furthermore, HBZ mRNA levels correlate with PVL and clinical status in HAM/TSP patients (Saito et al., 2009; Leal et al., 2018). This association is partially explained by unique functions of HBZ mRNA distinct from HBZ protein (Shiohama et al., 2016): Untranslated HBZ mRNA (substitution of ATG start codon with TTG) induces T-cell expansion, while HBZ mRNA with silent mutations that interfere with the secondary structure of RNA does not promote proliferation. HBZ RNA likely does this via induction of E2F1 gene expression, which is associated with oncogenesis and increased proliferation (Satou et al., 2006). The low protein level of HBZ can also be explained by strong nuclear retention of HBZ mRNAs, suggesting that the mRNAs might function as non-coding transcripts (Rende et al., 2011). Aberrant proliferation and expression of genes associated with cell cycle in HAM/TSP (Table 2) could also be related to the interplay of HBZ and Tax with host machinery.

**TABLE 2:** Alteration of transcriptomic profile of cellular pathways in HAM/TSP.

**Pathway/Process**	**Gene/Protein**	**Alteration (vs. ACs)**	**Tissue/Cells**	**References**
Cytokine production and signaling	STAT1*	Increase	Monocytes, B-cells, Neutrophils, CD8 T-cells	Tattermusch et al., 2012
		Decrease	PBMCs	Mozhgani et al., 2019
	ANKRD22	Increase	Whole Blood	Tattermusch et al., 2012
	GBP1	Increase	Whole Blood	Tattermusch et al., 2012
	GBP5	Increase	Whole Blood	Tattermusch et al., 2012
	EPSTI1	Increase	Whole Blood	Tattermusch et al., 2012
	P2RY14	Increase	Whole Blood	Tattermusch et al., 2012
	FVGR1B	Increase	Whole Blood	Tattermusch et al., 2012
	WARS	Increase	Whole Blood	Tattermusch et al., 2012
	IL15	Increase	Whole Blood	Tattermusch et al., 2012
	IRF1	Decrease	CD4 T-cells	Mozhgani et al., 2018
	VEGFA	Increase	CD4 T-cells	Mozhgani et al., 2018
	SMAD2	Decrease	CD4 T-cells	Mozhgani et al., 2018
	IFIT2	Increase	CD4 T-cells	Mozhgani et al., 2018
	IL23A	Decrease	CD4 T-cells	Mozhgani et al., 2018
	Cyp4f2	Decrease	CD4 T-cells	Pinto et al., 2013
	Mab21L2	Decrease	CD4 T-cells	Pinto et al., 2013
	IL27	Decrease	CD4 T-cells	Pinto et al., 2013
	TNFR	Decrease	Whole Blood	Youssefi et al., 2020
	GBP2	Increase	PBMCs	Fukutani et al., 2019
Cell cycle, apoptosis, and senescence	SHC1	Increase	CD4 T-cells	Mozhgani et al., 2018
	CCNA2	Increase	CD4 T-cells	Mozhgani et al., 2018
	CDC16	Decrease	CD4 T-cells	Mozhgani et al., 2018
	CDK6	Decrease	CD4 T-cells	Mozhgani et al., 2018
	Morf4	Decrease	CD4 T-cells	Pinto et al., 2013
	NAP1L1	Decrease	CD4 T-cells	Pinto et al., 2013
	SC02	Increase	Whole Blood	Tattermusch et al., 2012
	CD95	Increase	Whole Blood	Tattermusch et al., 2012
	BCL2	Decrease	Whole Blood	Youssefi et al., 2020
	CASP3	Decrease	Whole Blood	Youssefi et al., 2020
	CASP8	Increase	Whole Blood	Youssefi et al., 2020
T-cell development and differentiation	MYH11	Increase	CD4 T-cells	Mozhgani et al., 2018
	PPP2CA	Decrease	CD4 T-cells	Mozhgani et al., 2018
	ITM2B	Decrease	CD4 T-cells	Pinto et al., 2013
	SUM02	Decrease	CD4 T-cells	Pinto et al., 2013
	P2RX7	Increase	CD4 T-cells	Pinto et al., 2013
	PABPC3	Decrease	CD4 T-cells	Pinto et al., 2013
	CRACR2B	Decrease	CD4 T-cells	Pinto et al., 2013
Chromatin and gene expression regulation	hnPvNPAl	Decrease	CD4 T-cells	Pinto et al., 2013
	Scrt2	Decrease	CD4 T-cells	Pinto et al., 2013
	XP06	Decrease	CD4 T-cells	Pinto et al., 2013
	Histlh3i	Decrease	CD4 T-cells	Pinto et al., 2013
	Histlh3b	Decrease	CD4 T-cells	Pinto et al., 2013
	Histlh3e	Decrease	CD4 T-cells	Pinto et al., 2013
DNA damage response	ATM	Increase	CD4 T-cells	Mozhgani et al., 2018
	MSH2	Increase	CD4 T-cells	Mozhgani et al., 2018
	FEN1	Decrease	CD4 T-cells	Mozhgani et al., 2018
Glucocorticoid signaling	ANXA1	Decrease	CD4 T-cells	Mozhgani et al., 2018
	PTGES3	Decrease	CD4 T-cells	Pinto et al., 2013
TCR signaling and antigen presentation	FGFR1	Increase	CD4 T-cells	Mozhgani et al., 2018
	PTK2	Decrease	CD4 T-cells	Mozhgani et al., 2018
	TAP1	Decrease	PBMCs	Mozhgani et al., 2019
	PSMB8	Increase	PBMCs	Mozhgani et al., 2019
Cell migration	TIMP1	Decrease	CD4 T-cells	Mozhgani et al., 2018
	PXN	Increase	CD4 T-cells	Pinto et al., 2013
	CXCR4	Decrease	CD4 T-cells	Mozhgani et al., 2018
		Increase*		Pinto et al., 2013
Intercellular adhesion	FN1	Decrease	CD4 T-cells	Mozhgani et al., 2018
	BTN2A1	Decrease	CD4 T-cells	Pinto et al., 2013
	LGALS1	Decrease	CD4 T-cells	Pinto et al., 2013
Metabolism	ARF1	Decrease	CD4 T-cells	Mozhgani et al., 2018
	ATP5e	Decrease	CD4 T-cells	Pinto et al., 2013
	GPT	Decrease	CD4 T-cells	Pinto et al., 2013
	MCAT	Decrease	CD4 T-cells	Pinto et al., 2013
	GAPDH	Decrease	CD4 T-cells	Mozhgani et al., 2018
	Nacap1	Decrease	CD4 T-cells	Pinto et al., 2013
	TRXR	Decrease	Whole Blood	Youssefi et al., 2020
Cell-mediated lysis	GZMA	Increase	CD4 T-cells	Pinto et al., 2013
	PRF1	Increase	CD4 T-cells	Pinto et al., 2013
	FasL	Increase	CD8 T-cells	Kawahigashi et al., 1998
RNA degradation (Rnase III)	DICER1	Decrease	CD4 T-cells	Pinto et al., 2013

HTLV-1 affects gene expression in several non-mutually exclusive pathways in host. Genes with unknown function in the target cells were not included in the table. The data might not reflect changes in gene expression of infected cells; Pronounced modification in expression of genes related to T-cell differentiation, cytokine signaling, and TCR signaling demonstrates a hyporesponsive inflammatory profile in T-cells. *Change is accompanied by reduction in protein synthesis.

## HBZ and Tax in Pathogenesis of HTLV-1 Related Diseases

While the significance of PVL in determining the course of HTLV-1 infections and transformation of ACs to ATLL and/or HAM/TSP patients is already determined (Nagai et al., 1998; Iwanaga et al., 2010), the role of HBZ and Tax expression and their balance in the outcome of HTLV-1 infection is also strongly suggested by comparing their levels between ACs, HAM/TSP, and ATLL patients. Relative expression of Tax in ATLL patients is less than ACs and HAM/TSP patients (Furukawa et al., 1995; Yamano et al., 2002; Araya et al., 2011). Conversely, HBZ mRNA expression was found to be significantly lower in HAM/TSP patients compared to ATLL patients. However, a normalized comparison of HBZ mRNA levels (HBZ mRNA/Proviral DNA) did not vary between groups (Saito et al., 2009), suggesting this correlation in peripheral blood cells is based on PVL rather than increased HBZ transcription. Other studies also demonstrate that HBZ protein expression is correlated with PVL and not with host HBZ antibody nor HBZ mRNA levels (Shiohama et al., 2016). All things considered, the conflicting values in different studies may not represent any true correlation since the interdependent expression of Tax and HBZ are modified by various factors both *in vitro* and *in vivo* (Kulkarni and Bangham, 2018) that would be transiently affected by certain study circumstances. Moreover, HBZ mRNA levels in peripheral blood of HAM/TSP patients correlated with disease severity and neopterin; a marker of cellular immune response induced by Th1 (Saito et al., 2009). Furthermore, HBZ is primarily visualized in cytoplasm of CD4+ T-cells in HAM/TSP patients, in contrast to ATLL patients in which HBZ is located in the nucleus (Baratella et al., 2017; Forlani et al., 2019). Moreover, transgenic models with HBZ gene demonstrate inflammatory diseases resembling those found in infected individuals (Satou et al., 2011). Finally, higher affinity of HLA class 1 binding to HBZ but not Tax is associated with a lower PVL and more effective viral control and therefore, reduced risk of developing HAM/TSP (Macnamara et al., 2010). The roles of Tax and HBZ in infection and immune response will be discussed further in the section on viral and cellular interactions.

## p12 and p8

Open reading frame I of pX region in HTLV-1 viral genome encodes p12 protein which can be cleaved to produce p8 protein (Sarkis et al., 2019). The p12 protein is primarily localized in the Golgi apparatus and endoplasmic reticulum, while p8 interacts with TCR in immunological synapses in plasma membranes, reducing MHC-II expression and antigen presentation, respectively (Sarkis et al., 2019), contributing to HTLV-1 evasion of immune response. p12/p8 also have direct role in activation and proliferation of infected T-cells: Infected T-cells with HTLV-1 show spontaneous proliferation in the absence of IL-2 *in vitro* due to constitutive activation of JAK-STAT pathway in HTLV-1 infected cells (Migone et al., 1995). However, the role of p12/p8 in this pathway isn’t the direct phosphorylation of JAK/STAT proteins, but rather prevention of transport of IL-2R to the plasma membrane via binding with β and γc chains of the immature IL-2R, lowering IL-2R count on the cell surface (Mulloy et al., 1996; Sarkis et al., 2019). p8 also induces nanotubes in infected cells, facilitating transmission of viral particles and proteins to uninfected cells (Van Prooyen et al., 2010b).

## p30 and p13

Both p30 and p13 are encoded by open reading frame II in the pX region (Van Prooyen et al., 2010a). The nucleolus residing protein p30 is reported to be involved in viral latency, gene regulation, and cell cycle progression. p30 contributes to evasion of immune system surveillance by binding to tax/rex mRNA, facilitating its nuclear retention and reduced expression in infected cells. p30 also interferes with the immune response by downregulating TLR4 and disrupting interferon signaling via interacting with transcription factor PU.1 (Moles et al., 2019).

p13 is predominantly localized in the inner membrane of mitochondria of infected cells, where it increases the activity of electron transport chain and production of reactive oxygen species. Increased ROS synthesis in infected cells results in either T-cell activation and proliferation or apoptosis depending on the ROS setpoint in each cell. Nuclear p13 has also been demonstrated to decrease Tax interaction with CREB binding protein and viral transcription (Omsland et al., 2020). Therefore, p13 seems to contribute to viral latency and clonal expansion by inducing apoptosis in transformed cells and favoring proliferation of resting T-cells, respectively.

## Cell-To-Cell Viral Dissemination

The entry of virions into host cells and fusion process of viral particles with cell membrane is mediated by the surface unit of HTLV-1 receptor, gp46. The surface unit forms complexes with glucose transporter 1 (GLUT1), Heparan sulfate proteoglycan (HSPG), and neuropilin 1 (NRP-1) on target cells to initiate envelope fusion with the plasma membrane, leading to the release of viral core into cytoplasm (Soltani et al., 2019). The viral RNA is then reverse-transcribed into a double-stranded DNA and is integrated into the host genome.

As mentioned before, cell-free virions have diminished infectivity for host cells aside from dendritic cells (Alais et al., 2015). In addition, even though GLUT1 expression occurs in all tissues (Coskun and Sutton, 2005), the target receptor of HTLV-1 alone cannot adequately describe the viral distribution in host. HTLV-1 infectivity is limited to these cells *in vivo* with CD4+ T-cells comprising 95% of infected cells: CD4+ or CD8+ T-cells, B lymphocytes, monocytes, macrophages, dendritic cells (DC), glial cells, megakaryocytes, sweat gland epithelia, salivary glands, vascular endothelial cells, and mammary glands (Hoshino, 2012). These facts raise the question about the method of viral spread between host cells, which, as we will see later on, are also critical steps in HTLV-1 related disease pathogenesis.

## Virological Synapse and Extracellular Viral Assemblies

**FIGURE 2:**
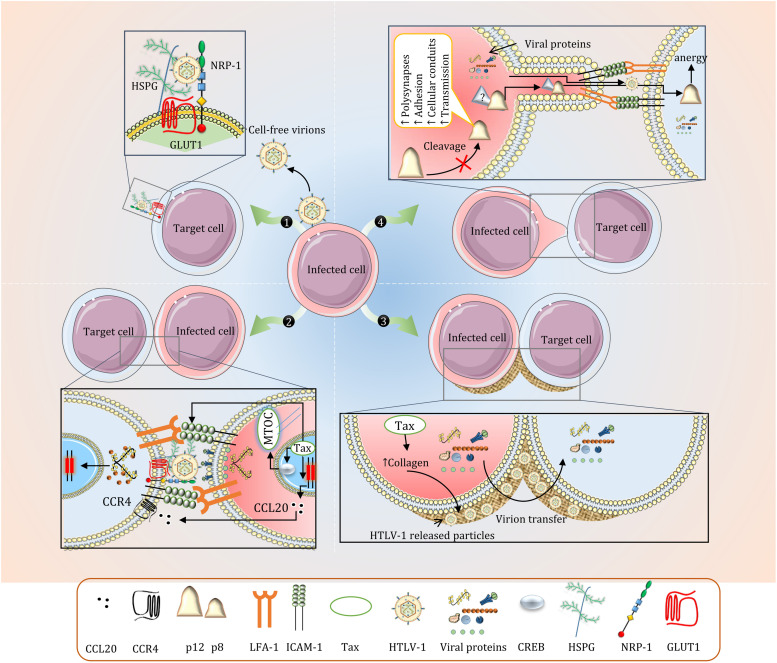
Intercellular Transmission of HTLV-1. (1) Cell-free virions are rarely produced in HTLV-1 infection and these particles are almost non-infectious (only 1 in 10^5^ to 10^6^). The fusion process is mediated by GLUT1, NRP-1, and HSPG. Other cell adhesion molecules including ICAM-3 and VCAM have also been associated with HTLV-1-induced cell fusion. (2) Viral transfer via Virological synapse (VS). Polarization of MTOC and expression of ICAM-1 and CCL22 is enhanced by Tax. While VS formation is primarily mediated by ICAM-1 and LFA-1, blocking CCL22 is shown to significantly reduce VS formation and viral transmission in CD4+ T-cells. (3) Concentrated viral particles in the extracellular assemblies is efficiently transferred to other cells upon contact. (4) The precise mechanisms regarding p8-mediated cellular conduits are not well understood. p8 in recipient CD8+ T-cells induces T-cell anergy which reduces effective CTL response.

The virus utilizes and upregulates cell-cell adhesion molecules for intercellular propagation and heavily relies on intimate cell-cell contact and cytoskeletal remodeling (Igakura et al., 2003). Viral transmission mainly occurs in close cell-to-cell contacts named Virological synapse (VS). VS is the result of tax mediated interaction between intercellular adhesion molecule 1 (ICAM-1) on infected cells with lymphocyte function-associated antigen 1 (LFA-1) on target cells accompanied by the polarization of microtubule organizing center (MTOC) inside the infected cells toward the target cell (Nejmeddine et al., 2009; Gross and Thoma-Kress, 2016). Tax promotes VS formation through upregulation of ICAM-1 on HTLV-1 infected cells, accumulation of Tax in microtubule organizing center, recruitment of fascin bundling protein, and CREB signaling of nuclear Tax (Banerjee et al., 2007a; Gross and Thoma-Kress, 2016; Gross et al., 2016). Cell-cell contact and viral transfer may also be improved by clustering of NRP-1 in VS similar to immunological synapses formed between DCs and T-cells (Tordjman et al., 2002). Upon polarization of MTOC, viral proteins at the cell cortex are released toward the cell contact. Viral components are then visualized inside target cells, demonstrating a successful viral transfer (Figure 2; Pais-Correia et al., 2010).

The upregulation of adhesion molecules also facilitates concentrated virion transfer in biofilms. These viral assemblies are formed on the cell surface when HTLV-1 released particles are trapped in extracellular matrix components primarily made up of a carbohydrate-rich structure containing sialyl-LewisX and heparan sulfate proteoglycans (Pais-Correia et al., 2010). Collagen was also observed in these assemblies, which is overexpressed in HTLV-1 infected cell lines in a tax-specific fashion (Figure 2; Munoz et al., 1995; Pais-Correia et al., 2010). This form of viral transfer has yet to be reproduced *in vivo*.

In many aspects, VS is advantageous for HTLV-1 persistence inside the host. The localized release of virions in synaptic cleft increases the efficiency of transmission while avoiding immune system recognition. VS also elucidates preferred tropism of HTLV-1 for CD4+ T-cells (Gross and Thoma-Kress, 2016), as ICAM-1 ligands such as LFA-1 are expressed in CD4+ T-cells. However, It can be speculated that viral spread using VS is limited in later stages of the disease as infected CD4+ T-cells have reduced MHC-I, ICAM-1, and ICAM-2 levels and increased LFA-1, probably due to p12/p8 effect on adhesion molecules (Banerjee et al., 2007a). It can be postulated that this may contribute to the phenomenon regarding the establishment of unique clones early in HTLV-1 infection and dominance of expansion of the existing clones in the chronic stage (Bangham, 2018; Bangham et al., 2019). This observation can also be interpreted as an attempt to escape the NK cell ICAM-mediated lysis of low MHC-I expressing cells (Topham and Hewitt, 2009). The reader is referred to other articles for a list of host cell proteins implicated in HTLV-1 transmission (Gross and Thoma-Kress, 2016).

## Cellular Conduits and Extracellular Exosomes

HTLV-1 is also transmitted via p8-induced cellular conduits *in vitro*. These conduits are thin-membrane extensions of infected cells appearing as an extended VS. While clustering of LFA-1 on the surface of infected cells seems to contribute to the formation of conduits, the precise mechanism for this process is not understood. These cellular conduits may also lead to CTL anergy and viral persistence by transferring p8 to CTLs (Van Prooyen et al., 2010b). The absence of p8 in several viral strains undermines the importance of these conduits in viral transfer.

Unilamellar exosomes released from infected cells during HTLV-1 are generally not infectious, but they may indirectly modulate infectivity by promoting adhesion molecule on recipient cells (Pinto et al., 2019). These extracellular vesicles contribute to the persistence of inflammation in CNS, which is further elaborated in the relevant sections (go to Central Nervous System section).

## Viral and Cellular Interactions in the Periphery

**FIGURE 3:**
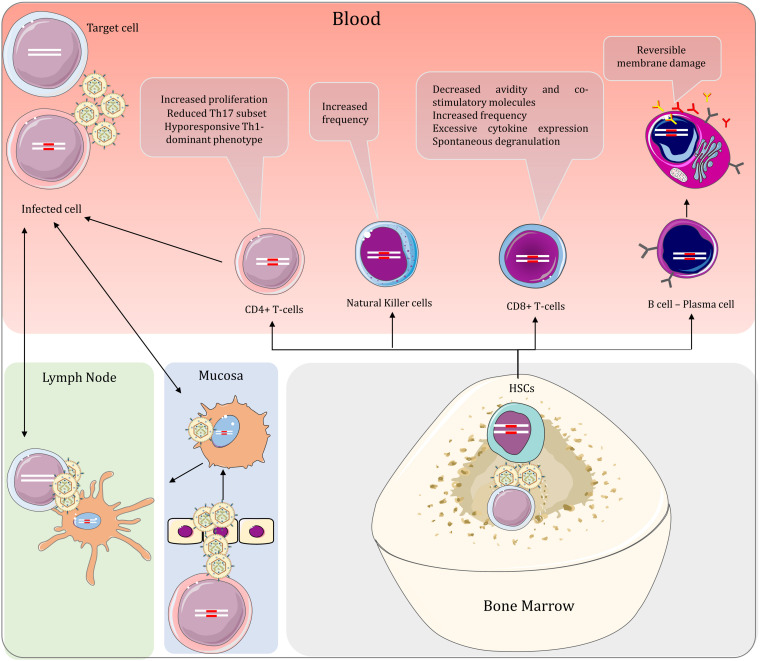
Peripheral interactions of HTLV-1 and the host in HAM/TSP and the effect of the virus on the lymphoid lineage cells. When the virus is acquired via mucosal infection, the dendritic cells spread the virus to immune cells in lymph nodes and the peripheral blood. Consequently, infected T-cells can also infect dendritic cells, spreading the virus bidirectionally between T-cells and dendritic cells in the periphery. HTLV-1 can also be delivered to hematopoietic stem cells in the bone marrow. These cells give rise to all other blood cells, including both myeloid and lymphoid lineages. By virtue of vertical transmission, proviral DNA is found in cells of both lineages, regardless of productive infection. The lymphoid lineage cells are studied more extensively in the context of HTLV-1 infection and are associated with prominent changes in HTLV-1-related diseases.

For HTLV-1 to enter the host, the virus must first pass through either the intestinal epithelium as in transmission of the virus through breastfeeding or reproductive tract mucosa as in sexual transmission. It is self-evident that acquiring the virus via blood products and organ transplantation bypasses the need to cross mucosal barriers. The virus can then be delivered to bone marrow and lymph nodes, where physiological hypoxia increases plus-strand expression (Kulkarni and Bangham, 2018). This helps to establish numerous clones before an effective anti-viral response could come into play (Figure 3).

HTLV-1 can either directly penetrate through a damaged epithelium following injury or utilize cellular-mediated pathways. It is also hypothesized that infected macrophages present in contaminated breast milk or semen can transmigrate through an intact epithelium (Pique and Jones, 2012). Alternatively, free HTLV-1 virions can cross the epithelial barrier via a transcytosis mechanism and infect human DCs beneath the epithelial barrier (Martin-Latil et al., 2012). There are discordant studies regarding the infection of epithelial cells, their role in virus entry, and whether they can be included as viral reservoirs in HTLV-1 infections (Zacharopoulos et al., 1992; Zacharopoulos and Phillips, 1997; Southern and Southern, 1998; Martin-Latil et al., 2012).

## Dendritic Cells

Nevertheless, after crossing the barrier, HTLV-1 must either interact with antigen-presenting cells (APCs) such as DCs or directly infect mucosal immune cells. This interaction can also occur without the need to cross the epithelial barrier through direct cell-cell contact of infected cells with patrolling cellular protrusion of DCs in the lumen (Rocamonde et al., 2019). HTLV-1 particles are highly infectious to these cells *in vitro* (Jones et al., 2008; Jain et al., 2009) and viral DNA is observed in DCs of HAM/TSP patients (Macatonia et al., 1992; de Castro-Amarante et al., 2016). It has also been demonstrated that DC depletion increases gag expression in HTLV-1 infected mouse CD4+ T-cells (Rahman et al., 2010). These findings advocate the critical role of DCs in the pathogenesis of HTLV-1 related diseases.

Dendritic cells are potent APCs that are essential in the immune response against viral infections. While different subtypes of DCs exist in blood (myeloid dendritic cells and plasmacytoid dendritic cells (pDC)) and mucosa (monocyte-derived dendritic cells), they all share their capability in presenting antigens to T-cells in lymphoid organs (Jones et al., 2008; Villadangos and Young, 2008). Infecting DCs is considered favorable for HTLV-1 as viral transmission can occur via viral capture and presentation of virions to resting T-cells in lymph nodes (Jones et al., 2008). Conversely, infected CD4+ T-cells can also infect DCs via VS (albeit to a lesser extent because of the low expression of LFA-1 on DCs compared to activated CD4+ T-cells), establishing a bidirectional viral transmission route (Shimauchi et al., 2019). This process is mediated by HTLV-1 binding to lectin DC-specific ICAM-3-grabbing non-integrin (DC-SIGN) and ICAM-2 and ICAM-3 on target cells (Ceccaldi et al., 2006). In these cells, antigen presentation by MHC-II is also associated with the spontaneous proliferation of lymphocytes (Macatonia et al., 1992). Counterintuitively, HTLV-1 infection reduces monocyte differentiation to DCs which is independent of monocyte infection (Nascimento et al., 2011). It is also reported that DC count is decreased in HAM/TSP (Azakami et al., 2009; Journo and Mahieux, 2011; Nascimento et al., 2011). This reduced differentiation is suspected to be due to increased IL-6 in sera of the patients (Nascimento et al., 2011). The implications of this finding, however, is not understood and requires further studies.

Dendritic cells are also known for their robust antiviral signaling with proinflammatory cytokines. Nevertheless, production of type 1 interferons by DCs provide modest benefit to patients possibly due to interference of tax with the intracellular antiviral signaling cascade (Bangham, 2018), reduced interferon secretion in the majority of DCs (Moles et al., 2019) and reduction in expression and phosphorylation of STAT1 in PBMCs (Mozhgani et al., 2019). Recently, the visualization of DCs in choroid plexus of normal rats has led to the hypothesis that DC access to CNS antigens may play an important role in inflammatory and immune-mediated disorders of CNS (McMenamin, 1999). However, the relevance of this discovery to pathogenesis of CNS inflammation in HAM/TSP remains to be investigated.

## Hematopoietic Stem Cells and Myeloid Lineage

All of DC subtypes are derived from a common hematopoietic stem cell (HSC) ancestor. HSCs are self-replicating, multipotent cells, which differentiate to lymphoid and myeloid lineages in bone marrow (Rocamonde et al., 2019). Identical proviral integration site between HSCs, PBMCs and neutrophils in HAM/TSP indicate that these cells are infected *in vivo* and give rise to HSC derived HTLV-1 infected lymphocytes, monocytes, DCs and other hematopoietic lineages and act as viral reservoirs in persistent infections (Grant et al., 2002; Yasunaga et al., 2016; Furuta et al., 2017). Intriguingly, whereas infected monocytes and pDC are visualized in the blood of HAM/TSP patients, both monocytes and pDC show high resistance to HTLV-1 infections in *in vitro* studies (Assil et al., 2019; Rocamonde et al., 2019). HTLV-1 infection of monocytes results in abortive infection by two mechanisms: SAMHD1 restriction factor, a dGTP-dependent deoxynucleotide triphosphohydrolase, reduces dNTPs to below the levels required for reverse transcription. Furthermore, SAMHD1 binding to viral intermediates leads to formation of complexes with STING DNA sensor to trigger establishment of an IRF3-Bax complex and subsequent apoptosis of the cell (Sze et al., 2013). SAMHD1 restricting properties are only observed in quiescent T-cells (Descours et al., 2012), explaining why some HTLV-1 proteins aim to activate T-cells. The pDC response against HTLV-1 is mediated by TLR-7 signaling and TRAIL death receptor (Olière et al., 2011; Rocamonde et al., 2019). Studies demonstrate that TLR-7 signaling is impaired by HTLV-1-induced miR146a; Nonetheless, HTLV-1 infected pDCs show a strong TLR7-dependent IFN-a response (Olière et al., 2011). The discrepant observations regarding monocyte infection in different studies and the resistance of activated and mature DCs to HTLV-1, unlike immature DCs, raise the possibility that these cells are primarily infected before differentiation and are not byproducts of *de novo* infections (Rizkallah et al., 2017; Rocamonde et al., 2019). Notwithstanding, infection of myeloid cell lines is less productive than their lymphoid counterpart (Grant et al., 2002), because Tax transactivation of plus-strand is limited in this cell lineage (Lepoutre et al., 2009).

While neutrophils seem to retain normal functions and cytokine profile in HTLV-1 infections (Bezerra et al., 2011), persistent infection of monocytes has been directly associated with the pathogenesis of HAM/TSP. Infected monocytes and macrophages can migrate to CNS during inflammation-induced blood-brain barrier permeability, delivering the virus to the CNS. HAM/TSP patients also show increased intermediate monocytes (CD14+CD16+) which are also the dominant monocyte subtype in producing Tumor necrosis factor (TNF), a major proinflammatory cytokine in HAM/TSP (Amorim et al., 2014). In addition, non-classical monocytes (CD4^*low*^CD16+) in HAM/TSP patients demonstrate an inflammatory phenotype evidenced by higher levels of CX3CR1 and HLA-DR compared to ACs (Enose-Akahata et al., 2012b). The role of these cells in pathogenesis of CNS is further investigated in the CNS section.

## CD4+ T-Cells

It is widely known that HTLV-1 is primarily found in CD4+ T-cells and proliferates by driving the cells to replicate (Asquith et al., 2007). HTLV-1 also modifies the immune response by increasing memory T-cells while reducing naïve T-cells (Yasunaga et al., 2001). This tendency is common between ACs and ATLL and HAM/TSP patients (Furukawa et al., 1992; Takemoto et al., 1994; Bangham, 2018). In HAM/TSP, HTLV-1 also decreases Th17 effector subset, further impairing host immunity (Leal et al., 2013). The imbalance in T helper system in HAM/TSP leads to a Th1 dominant immunophenotype accompanied by proinflammatory cytokines (IFN-γ, TNF-α, and IL-2) (Furuya et al., 1999; da Silva Dias et al., 2016; Nozuma and Jacobson, 2019). The resultant inefficient immune response is rather detrimental than being protective in individuals with HAM/TSP. The mechanism for these changes still remains ambiguous but is visualized by altered expression of genes in processes related to cell differentiation in CD4+ T-cells (Table 2). The function of CD4+ compartment of T-cells is not only dysregulated by the above but also by aberrant expression of other cell markers which is described below.

T-cell factor 1 (TCF 1) and lymphoid-enhancer binding factor 1 (LEF1) both act as transcription factors in Wnt pathway and are primarily expressed in immature T-cells. They antagonize tax transactivation of 5′LTR and may impair viral replication in Thymus and favor viral replication in TCF1/LEF1 low-expressing mature peripheral T-cells observed in HTLV-1 infection (Ma et al., 2015). Apparently, these factors are also reduced in HTLV-1 infection. Considering TCF1 role in preserving effector function of exhausted CD8 T-cells during chronic viral infection (Wang et al., 2019), examining this alteration might provide new insights in treatment of HTLV-1 related diseases. Intriguingly, higher expression of PD-1, another marker associated with T-cell exhaustion, along with loss of CD28, reflecting senescence, is also observed in both CD4+ and CD8+ T-cells in HAM/TSP and ATLL (Shimauchi et al., 2007; Saeed et al., 2020). Surprisingly, it seems that the PD-1 increase is associated with regulatory CD4+ T-cells (Tregs) rather than effector CD4+ T-cells as PD-1 expression in the latter is actually decreased (Leal et al., 2013). The loss of PD-1 on effector T-cells might reflect their active and non-anergic state observed in HAM/TSP.

The reason why HTLV-1 predominantly resides in CD4+ T-cells is not completely understood, yet can be explained by some of the recent discoveries such as the interaction of HBZ with THEMIS. The co-inhibitory receptor T-cell immunoglobulin and ITIM domain (TIGIT) exerts its anti-inflammatory properties via interaction with their intracytoplasmic immunoreceptor tyrosine inhibitory motif (ITIM) domain which subsequently interacts with a complex of SHP-2, Grb2, and THEMIS (Tanaka and Matsuoka, 2018). HBZ interaction with THEMIS inhibits IL-10 production induced by TIGIT that leads to unresponsive proliferation of cell. Knock-down of THEMIS essentially removes cytoplasmic HBZ demonstrating that THEMIS is required for cytoplasmic localization of HBZ (Tanaka and Matsuoka, 2018). Therefore, THEMIS might explain the productive infection of T-cells. CCR4 ligand CCL22 expression by Tax also attracts CCR4+ T-cells and may also contribute to VS formation with CD4+ T-cells (Hieshima et al., 2008). The role of APCs in delivering HTLV-1 to CD4+ T-cells should not be neglected either. Evidence suggests that the predominant presence of HTLV-1 in CD4+ T-cells rather than CD8+ T cells might be a post-infection event due to selective clonal expansion over time (Kannian et al., 2012).

## Regulatory CD4+ T-Cells

Regulatory T-cells (Tregs) are a unique subset of T-cells capable of suppressing antigen-specific responses by effector cells (Bangham and Toulza, 2011). Whereas these cells are generally identified as CD4+CD25+Foxp3+, both CD25- and Foxp3- cells with suppressive Treg functions have been demonstrated (Ohkura et al., 2013; Zhao et al., 2017). Furthermore, the transcriptional regulator Foxp3, which is implicated in the development of Treg functions, is expressed in some inflammatory subpopulations of CD4+ T-cells (Ohkura et al., 2013). Moreover, activation of T-cells (as seen with Tax) induces a transient expression of Foxp3 that is not accompanied by Treg functions (Bangham and Toulza, 2011). Therefore, the circumstances have misled the literature to assume that these CD4+CD25+Foxp3+ T-cells, which have been associated a Th1-like overproduction of IFN-γ and induction of proliferation of Tax-specific CD8+ T-cells in HAM/TSP, were originally Tregs (Araya et al., 2011). Nevertheless, CD4+CD25+CCR4+ T-cell population have been noted to be prime viral reservoirs of the virus in both ATLL and HAM/TSP patients (Yamano et al., 2004, 2009).

Tax and HBZ have been associated with the dysregulation of Foxp3 expression. HBZ induces TGF-β/Smad pathway to increase Foxp3+ T-cells in HBZ-transgenic mice (Satou et al., 2011; Zhao and Matsuoka, 2012), but also interact with Foxp3 to interfere with its DNA binding activity (Tanaka and Matsuoka, 2018). Furthermore, it has been shown that induced Tregs are increased in transgenic model with unstable Foxp3 expression (Yamamoto-Taguchi et al., 2013). Although controversial, it seems that the former effect of HBZ in increasing Foxp3 is nullified as Foxp3+ T-cells count is lowered in humans (Yamano et al., 2005; Araya et al., 2011). Moreover, it seems Foxp3+ Tregs are not terminally differentiated and can lose their Foxp3 expression and therefore convert to effector IFN-γ-producing T-cells (Yamamoto-Taguchi et al., 2013). HBZ dysregulation of Foxp3 pathway is confirmed by the fact that immune response of these T-cells in the transgenic model is not directed at HBZ but is attributed to intrinsic properties of HBZ-expressing cells (Yamamoto-Taguchi et al., 2013). Role of Tax in dysregulation of Tregs seems to oppose HBZ; Tax transactivation of OX40 (CD134), a costimulatory molecule from TNF receptor family and its ligand OX40L (gp34), induce proliferation of memory T-cells whereas suppressing the differentiation and activity of Tregs (Higashimura et al., 1996; Enose-Akahata et al., 2017). Tax also inhibits TGF-β pathway and expression of Foxp3, while it increases IFN-γ promoter activity (Sugata et al., 2012; Zhao and Matsuoka, 2012).

In spite of all the evidence, conflict exists whether loss of Foxp3 is associated with reprogramming of Tregs or Foxp3 loss in conventional T-cells (Miyao et al., 2012; Yamamoto-Taguchi et al., 2013) or whether Foxp3 expression is increased or decreased in HAM/TSP (Yamano et al., 2005; Oh et al., 2006; Hayashi et al., 2008; Toulza et al., 2008; Araya et al., 2011; Pinto et al., 2013; Bangham et al., 2015). Nevertheless, there is also the unanswered question whether this pattern is induced by direct infection of Tregs or a dysregulation in differentiation of infected HSCs. These controversies demand much more attention and must be scrutinized.

## CD8+ T-Cells and NK Cells

Despite carrying 5% of PVL (Melamed et al., 2015), CD8+ T-cells play a fundamental role in both neuropathology and viral latency in HAM/TSP. It is of note that HTLV-1 specific CTLs are significantly increased in PBMCs of HAM/TSP when compared to ACs. CD8+ T-cell response in peripheral blood of HAM/TSP patients and ACs is normally directed toward the plus-strand protein Tax (Elovaara et al., 1993). The amplitude of the response however, is increased in HAM/TSP. The increased frequency of these cells is associated with higher PVL and might suggest inefficiency in disease prevention (Kubota et al., 2000a,b; Wodarz et al., 2001; Leal et al., 2013). On the other hand, HTLV-1-specific CTL responses with higher efficiency lead to lower PVL and better viral control; This indicates the quality of CTL cell-mediated lysis and not the frequency of these cells to be a major determinant in anti-viral defense vs. HTLV-1 (Bangham and Osame, 2005; Bangham, 2009). This discovery is also evident in the fact that CD8+ T-cells of HAM/TSP patients have reduced co-stimulatory molecules compared to ACs in similar PVLs (Sabouri et al., 2008).

CD8+ T-cells show dysfunctions, some of which are uniquely observed in HAM/TSP (Enose-Akahata et al., 2008). Evidence suggests that aberrant function of these cells in HAM/TSP may be the result of various factors including: high antigenic stimulation, Tax dysregulation of cytokine loops (IL-2/IL-2R and IL-15/IL-15R), and selective expression of surface receptors (CD244) which ultimately results in reduction of T-cell avidity, spontaneous degranulation, excessive cytokine expression (IFN-γ, TNF, and IL-2) and subsequent damage to the host tissues (Kubota et al., 1998; Enose-Akahata et al., 2008, 2017; Sabouri et al., 2008; Kattan et al., 2009; Yasuma-Mitobe and Matsuoka, 2018; Nozuma and Jacobson, 2019). Indeed, PVL is significantly elevated in CSF of HAM/TSP patients compared to their peripheral blood (Puccioni-Sohler et al., 1999). CD14+ cells (Mononuclear phagocytes) may also have a role in dysregulation of CTLs. Spontaneous degranulation and IFN-γ release of CD8+ T-cells which has been linked to higher PVL and Tax mRNA expression is observed when these cells are cocultured with IL-15-expressing-CD14+ cells and not CD4+ T-cells (Enose-Akahata et al., 2008). CTLs role in pathogenesis of HAM/TSP in CNS which will be discussed later.

Similar to CD8+ cytotoxic cells, NK cells also show impairment in their function in form of continuous activation. This had resulted in spontaneous degranulation and hypo-responsiveness as well as increased IFN-γ expression in this cell lineage (Queiroz et al., 2019). These changes apparently reflect malfunction of cell-mediated immunity in HTLV-1 infection. The NK cell (CD56+) frequency is diminished in HTLV-1 infections and HAM/TSP (Azakami et al., 2009; Tattermusch et al., 2012; Amorim et al., 2019) (Although Some studies do not show a reduced count (Tattermusch et al., 2012; Saeed et al., 2020)) and NKT cells (CD56+/CD3+) demonstrate reduced proliferation when exposed to activation molecules (Azakami et al., 2009). NK cell receptors like NKG2D, KIR2DL2, NKp30, and CD137 have been shown to modulate immune response against HTLV-1 and confer anti-viral protection, but these findings can also be attributed to CD8+ T-cells due to overlapping surface receptors and functionalities with NK cells (Bangham and Osame, 2005; Hudspeth et al., 2013; Bangham, 2018; Boelen et al., 2018; Amorim et al., 2019; Wu et al., 2019).

## B-Cells and Antibodies in HAM/TSP

The focus of studies regarding B-cells is mainly directed at their capacity to create antibodies as antibody secreting cells (ASCs). Older studies have stated that attempts in infection of B-cells with HTLV-1 had been unsuccessful (Richardson et al., 1990), thus studies regarding the importance of infection of B-cells in HTLV-1 related diseases are scarce (Longo et al., 1984), even though newer studies have demonstrated clonal expansion of HSCs has yielded infected B-cells (Furuta et al., 2017). Be that as it may, B lymphocytes themselves are not primarily infected by HTLV-1; but still, these cells show pathologic changes in HAM/TSP. Both circulating and memory B-cells show reversible membrane damage in which phosphatidylserine residues are exposed at outer membrane leaflet (Furukawa et al., 2000). These cells revert to their normal phenotype over time *in vitro*. The reason and significance for this disruption is not well understood.

Antibody (Ab) responses exist in viral infections of CNS and HTLV-1 is no exception. Indeed, the laboratory diagnosis of HAM/TSP is based on presence of anti-HTLV-1 Abs in CSF (Yamano and Sato, 2012). Humoral immune responses against Gag and Env are significantly increased in blood of HAM/TSP vs. other HTLV-1 infected individuals; whereas anti-Tax and anti-HBZ Ab levels are similar in HTLV-1 infected subjects (Enose-Akahata et al., 2012a; Shiohama et al., 2016). These Abs appear to provide some levels of protection against HTLV-1 in experimental studies (Tanaka et al., 1994; Saito et al., 2014). Intrathecal antibodies also inversely correspond with a higher PVL and a worse prognosis (Puccioni-Sohler et al., 2007). Despite their protective functions, endogenous antibodies are considered to be ineffective in virus control in humans (Kitze et al., 1996; Cook et al., 2013).

## Blood Brain Barrier Dysregulation and Virus Entry to CNS

**FIGURE 4:**
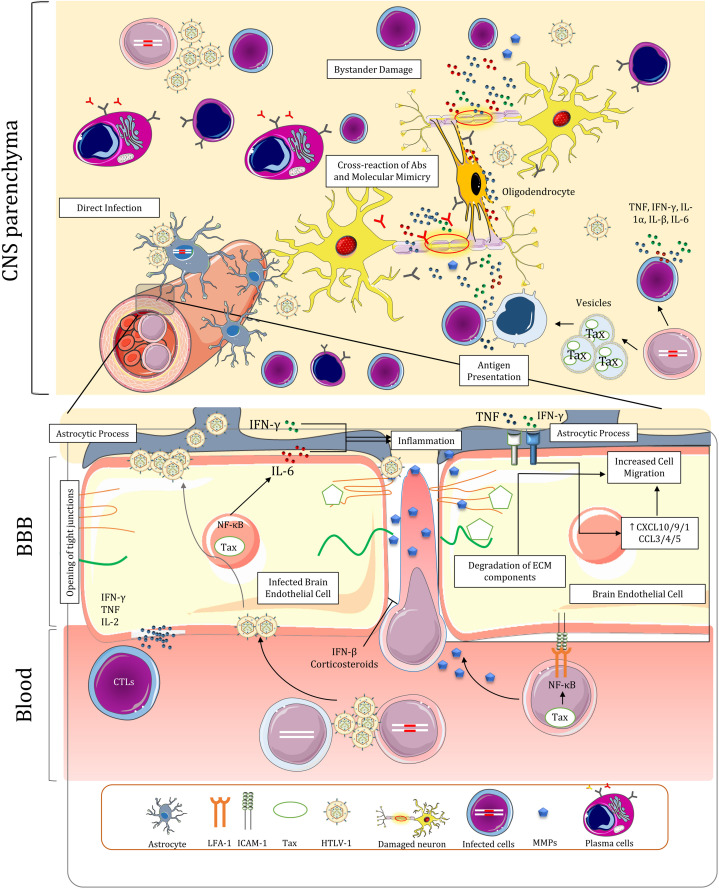
Blood-brain barrier dysregulation, infiltration of immune cells, and proposed mechanisms for the pathogenesis of HAM/TSP in the central nervous system. The BBB is dysregulated due to multiple factors in HAM/TSP, leading to persistent inflammation in the CNS. The inflammation resulting from the presence of the virus and the immune response establishes a positive feedback loop that furthrer disrupts BBB and attracts other immune cells, including infected T-cells.

HAM/TSP shares similarities in many aspects with MS (Puccioni-Sohler et al., 2007; Azodi et al., 2017). In this regard, Inflammatory cells must cross the normally intact blood-brain barrier (BBB) to react with CNS parenchyma. Both diseases show degradation of BBB components and inflammation-mediated increase in its permeability (Umehara et al., 1998; Liebner et al., 2018). BBB dysfunction can occur due to numerous factors HAM/TSP. HTLV-1 methods of access to CNS can be roughly divided into two categories: Direct virus interaction with components of BBB and non-BBB-cell-mediated virus entry to CNS (Figure 4).

Brain endothelial cells (BECs) have been shown to be directly involved in BBB dysregulation. It has been demonstrated that HTLV-1 can infect both rat and human (BECs) via virus-induced cell-cell fusion with infected lymphocytes (Uchiyama, 1997; Afonso et al., 2008). Infection of these cells can then lead to release of viral particles at their abluminal surface and infection of astrocyte foot processes which surround brain capillaries, effectively delivering the virus to CNS (Romero et al., 2000). Infected BECs no longer maintain resistance to molecular diffusion and cellular migration *in vitro* (Afonso et al., 2008). The changes include increased transcytosis and opening of tight junctions between BECs (Romero et al., 2000). These cells can also be targets of CTLs and autoantibodies *in vivo* resulting in temporary disruption of BBB (Romero et al., 2000; Afonso et al., 2008). The migration of inflammatory cells and their interaction with CNS components also induce a cytokine loop that further disrupts BBB and promotes cell migration. Tax-mediated activation of NF-κB in BECs lead to increased IL-6 secretion (Rott et al., 1993). High IFN-γ expression in the context of viral infection negates IL-6 anti-inflammatory effects via inhibiting STAT3-dependent IL-6 signaling (Bluyssen et al., 2010). This may contribute to cell-mediated entry by inducing focal inflammation and attracting nearby immune cells (Rott et al., 1993). Tax induction of NF-κB and STAT pathway may also increase CD4+ T-cell recruitment via the gateway reflex; however, the thoracic distribution of lesions in spinal cord of patients does not correspond with lumbar entry of cells in this pathway (Kamimura et al., 2019). Depletion of Th17 cells and negligible concentration of CCL20 in HAM/TSP (Ando et al., 2013; Sabharwal et al., 2014), which are both implicated in this reflex, might be responsible for lack of prominent migration in lumbar levels of spinal cord. Lastly, IFN-γ and TNF secretion by immune cells in CNS directly affects the cytokine profile of BECs by upregulating CXCL10, CXCL9, CX3CL1, CCL3, CCL4, and CCL5 expression and redistributing CCL3 to apical surface of these cells all of which ultimately results in further cell migration (Larochelle et al., 2011; Ando et al., 2013).

Upregulation of adhesion molecules by HTLV-1 might facilitate transmigration of immune cells across the BBB. Increased LFA-1 expression on surface of infected lymphocytes may amplify ICAM/LFA-1 interactions with BECs and promote adhesion of HTLV-1 infected cells to these cells (Romero et al., 2000). Endothelial cells in vicinity of spinal cords lesions express higher levels of Vascular cell adhesion molecule 1 (VCAM-1) which may interact with VLA-4 on immune cells to facilitate diapedesis (Umehara et al., 1996, 1998). However, this interaction was described to be of less importance in later studies as VCAM-1 increase is only observed following long-term cytokine stimulation and VLA-4 is decreased in isolated T-cells from HAM/TSP subjects (Romero et al., 2000). Activated Leukocyte Cell Adhesion Molecule (ALCAM) which was reported to be implicated in transmigration of CD4+ T-cells and MPs in MS (Lyck et al., 2017), is increased in HTLV-1 infected Tregs in HAM/TSP (Curis et al., 2016). Finally, OX40 and OX40L have been demonstrated to have a role in adhesion of T-cells to non-brain endothelium in ATLL (Romero et al., 2000). The role of this interaction in HAM/TSP has yet to be determined.

HTLV-1 infection can also modify gene expression in cells that might favor cell migration through the BBB. Gene expression in several pathways related to cell migration and ECM modifications are altered in CD4+ T-cells of HAM/TSP patients (Table 2). Paxillin (PXN) and Cytoskeleton organizer collapsin response mediator protein 2 (CRMP2) which are involved in cell migration pathway are increased in CD4+ T-cells of HAM/TSP patients and HTLV-1 infections, respectively (Varrin-Doyer et al., 2012; Pinto et al., 2013). Furthermore, Matrix metalloproteases (MMPs) which degrade ECM components, have been implicated in the pathogenesis of HAM/TSP by dysregulating BBB function and having a deleterious effect on ECM composition of CNS that leads to neuronal apoptosis (Umehara et al., 1998; Lakhan et al., 2013). MMP expression dysregulation is conspicuous due to observations demonstrating higher expression of MMP gelatinases (MMP-2 and MMP-9) in blood and CSF of HAM/TSP patients and downregulation of Tissue inhibitor of metalloproteinases 1 (TIMP-1) in CD4+ T-cells (Umehara et al., 1998; Mozhgani et al., 2018). Tax-mediated NF-κB activation may be responsible for elevated MMP-9 expression in HTLV-1-infected T-cells (Mori et al., 2002). Inactive chronic lesions of spinal cords of contained fewer MMP-2-positive or MMP-9-positive mononuclear cells than active-chronic lesions (Umehara et al., 1998). In light of the above, MMPs can be regarded as a key factor in bystander damage of CNS resident cells explained later.

IFN-β and corticosteroids, which are used both in MS and HAM/TSP therapy, have restorative effects on BBB function and reduce cell migration (Umehara et al., 1998; Bangham et al., 2015; Sweeney et al., 2019). Glucocorticoid signaling pathway is dysregulated in HAM/TSP which is characterized by downregulation of expression of Annexin A1 and Prostaglandin E synthase 3 in CD4+ T-cells (Mozhgani et al., 2018). The treatments in this regard, however, have shown modest improvement and lose efficacy over the course of disease (Nakagawa et al., 1996; Bangham et al., 2015). Nevertheless, the efficacy of corticosteroids in amelioration of the symptoms outweigh their adverse effects. Transient improvements in treatment with pulsed methylprednisolone and higher long term function with prednisolone has been observed (Araujo et al., 2020). The existing data is insufficient to recommend for other proposed treatment regimens for HAM/TSP such as IFN therapy, blood purification (lymphocytapheresis and plasmapheresis), Vitamin C, antiretroviral drugs, cyclosporine (Bangham et al., 2015), pentosan polysulfate sodium, pentoxifylline, and prosultiamine (Araujo et al., 2020).

## Central Nervous System

The neuropathology of HAM/TSP is marked by perivascular infiltrates of PBMCs, chronic demyelination and axonal loss throughout the CNS (Aye et al., 2000; Bangham, 2018; Nozuma and Jacobson, 2019). Lesions are primarily located in white matter of lower thoracic cord of the patients and infiltrating cells mainly comprise T-cells (Iwasaki, 1993; Wu et al., 1993; Matsuoka et al., 1998), where viral and cellular interactions result in spinal manifestations of HAM/TSP. Evidence of neurodegeneration and metabolic changes also exists in brains of both ACs and to a greater extent in HAM/TSP patients (Dimber et al., 2016; Schütze et al., 2017). The inflammation in brain is accompanied by global cognitive impairment and executive dysfunction in HAM/TSP patients (Champs et al., 2019). The exact mechanisms of neurodegeneration and myelin loss in CNS are not understood, but three putative mechanisms have been proposed: (1) direct infection of CNS resident cells; (2) bystander damage to resident cells of CNS during chronic inflammation; and (3) Cross-reaction of Abs and molecular mimicry (Figure 4; Olière et al., 2011; Bangham, 2018; Nozuma and Jacobson, 2019). The validity and contribution of these hypotheses to the pathology of HAM/TSP will be discussed in this section along with major histopathological and functional changes of CNS cells.

## Inflammatory Response of Immune Cells in CNS

Unlike monocytes and macrophages (Ginhoux and Prinz, 2015), activated T-cells are recruited to CNS under normal conditions (Hickey et al., 1991; Lehmann, 1998). It can be hypothesized that Infected lymphocytes could cross the barrier in this manner and express HTLV-1 proteins in CNS parenchyma. The presence of HTLV-1 in CNS induces inflammation and cytokine release by the resident CNS cells and migratory immune cells characterized by increased levels of TNF, IFN-γ, IL-1α, IL-β, and IL-6 in CSF and spinal cords of HAM/TSP patients (Umehara et al., 1994; Banerjee et al., 2007b; Champs et al., 2019). The ensuing recruitment of inflammatory cells from peripheral blood induces a vicious cycle of inflammation which is detrimental to CNS resident cells (Umehara et al., 1993; Furuya et al., 1999; Banerjee et al., 2007b). Over the course of inflammation, the number of CD4+ T-cells subsides in lesions (Umehara et al., 1993), probably because they are eliminated by HTLV-1-specific CD8+ T-cells. Indeed, widespread Fas+ T-cells are colocalized with apoptotic T-cells in active lesions (Umehara et al., 2002) while soluble Fas ligand is increased in CSF of HAM/TSP patients (Saito et al., 1999), suggesting increased Fas-mediated apoptosis of CD4+ T-cells in CNS. It is unclear whether the apoptotic T-cells are predominantly the result of immune response or FasL expression by astrocytes and BECs as part of physiological immune tolerance mechanism in CNS (Volpe et al., 2016). Distinct Fas genotypes may be associated with higher PVL and worse disease outcome in HTLV-1 infected carriers (Vallinoto et al., 2017).

Persistent inflammation in the CNS is not limited to the response against virus-infected cells. HTLV-1 infected cells release non-infectious extracellular vesicles containing Tax. It is noteworthy that CSF localization of these vesicles appear to be specific to HAM/TSP patients and is not normally observed in ACs (Anderson et al., 2018). Uptake of these exosomes by uninfected cells upregulates ICAM-1 and increases their susceptibility to VS-mediated infection (Pinto et al., 2019). Acquisition of Tax via these vesicles may also turn the uninfected cells to a potential target of cytotoxic degranulation of CTLs (Anderson et al., 2018). It can be surmised that high cytotoxicity against Tax+ monocytes *in vitro* (Matsuura et al., 2017) is the result of this interaction; since monocytes are considered resistant to HTLV-1 infection (Rocamonde et al., 2019). Moreover, perivascular macrophages can act as APCs (Hickey and Kimura, 1988; Lepoutre et al., 2009); presenting Tax epitopes to T-cells by scavenging apoptotic bodies of infected cells and the exosomes. It is unclear whether neurons or other CNS resident cells could acquire these vesicles; therefore, the extent of contribution of these exosomes to pathogenesis of HAM/TSP remains obscure (Pinto et al., 2019). One can assume that the antigen presentation, Tax uptake, and immune response of the large populations of infiltrating macrophages and microglia in CNS lead to the low-level inflammation in late-stage, chronic lesions, where infected CD4+ T-cells become sparse (Matsuura et al., 2017). This assumption is questionable owing to the fact that this activity is relative to the amount of HTLV-1 proviral DNA (Abe et al., 1999). These cells also seem to be inactivated during prolonged durations of the illness in chronic lesions (Abe et al., 1999).

Antibody secreting cells in the CSF are mainly involved in anti-Gag intrathecal antibody synthesis. These cells are not observed in ACs indicating a possible role of CSF ASCs in neuropathology of HAM/TSP (Enose-Akahata et al., 2018). Anti-Gag Abs were reported to cross-react with peroxiredoxin-1 (Prx1) and human transaldolase (TAL-H) while anti-Tax Abs react with heterogenous nuclear riboprotein A1 (hnRNPA1) (Banki et al., 1994; Levin et al., 2002; Jernigan et al., 2003; Kalume et al., 2004; Lee et al., 2008). However, the idea of molecular mimicry in CNS damage was later challenged by higher presence of hnRNPA autoantibodies in MS, similar antibody titer in other viral and neurodegenerative diseases, the absence of Tax in some HAM/TSP patients, and the fact that the expression of these genes is not restricted to CNS (Pulford et al., 1995; Ramirez et al., 2003; Yukitake et al., 2008; Fagerberg et al., 2014). It has been demonstrated that HAM/TSP IgG antibodies inhibit neuronal firing of nigral and pyramidal neurons but these immunoglobulins do not alter the viability of neurons during culture (Kalume et al., 2004). Nevertheless, the increase in barrier permeability and presence of antibodies in parenchyma may facilitate complement-mediated cell lysis (Ozden et al., 2002). The scale of protective and pathogenic functions of Abs in HAM/TSP is still largely not understood. In conclusion, there is a positive correlation between ASCs in CSF, Tregs, and PVL in patients (Enose-Akahata et al., 2018), but it is unclear if it translates to a unique immunopathological finding or being a consequence of neuroinflammatory milieu.

## HTLV-1 and CNS Resident Cells

HTLV-1 can both directly and indirectly impact the normal function of neurons and glial cells. While HTLV-1 is capable of infecting both neurons and glial cells *in vitro* (Watabe et al., 1989; Akagi et al., 1992), only astrocytes were shown to be infected by HTLV-1 in patients by *in situ* hybridization (Lehky et al., 1995; Lepoutre et al., 2009). Numerous studies have elucidated the prominent role of astrocytes in pathology of HAM/TSP (Méndez et al., 1997; Szymocha et al., 2000a,b; Akaoka et al., 2001; Ando et al., 2013). These cells maintain the normal function of neurons throughout the CNS. Dysregulation of this subtype of cells in HAM/TSP disrupts glutamate homeostasis which may lead to neuronal excitotoxicity (Sofroniew and Vinters, 2010). It is demonstrated that HTLV-1 infection may lead to apoptosis of glial cells *in vitro*, especially oligodendrocytes (Matute et al., 2006). Nonetheless, the impact of direct infection of CNS resident cells is considered to be minimal in neuronal damage (Bangham and Osame, 2005; Bangham, 2018).

The culmination of robust synthesis of proinflammatory cytokines by virtually every cell type present in CNS and the immune response of infiltrating cells during HTLV-1-induced inflammation is the bystander damage of resident cells in CNS (Bangham and Osame, 2005). Tax transduction to astrocytomas and oligodendrogliomas results in significant upregulation of proinflammatory cytokines including TNF, IL-1α, IL-1β, IL-6 (Banerjee et al., 2007b). NT2-N neuron-like cells also express TNF when exposed to Tax (Cowan et al., 1997). High concentration of TNF in active lesions sensitizes oligodendrocytes to apoptosis and induces secretion of MMPs in astrocytes which further disrupts myelin structure and BBB integrity and increases conversion of TNF-precursor to its active form (Giraudon et al., 1997; Banerjee et al., 2007b). High IFN-γ secretion by infected cells also induces CXCL10 expression in astrocytes which in turn recruits more infected cells to the area and prolongs the inflammatory response. Therefore, it appears that astrocyte responses seem to establish a positive feedback loop contributing to chronic CNS inflammation (Ando et al., 2013).

Despite numerous studies revolving around HTLV-1 myelopathy, data regarding neuronal pathogenesis in HAM/TSP is lacking. While the death of glial cells and lack of trophic support coupled by direct damage by free radicals, cytokines, and destructive enzymes are most likely the cause, the precise pathology of axonal degeneration remains unclear. Identifying the mechanism of neuronal damage may provide a major breakthrough in elucidating the course and pathogenesis of not only HAM/TSP but other inflammatory demyelinating diseases as well.

## Conclusion

Nearly 40 years of studying on HTLV-1 has yielded a myriad of findings concerning HTLV-1 structure and the effect of the virus on the human host. The study of retroviral clonality in HTLV-1 associated diseases has revealed that the integration site and transcriptional orientation of the provirus in the host genome and the selection force of host immune system against the clones lead to a signature pattern of clonality in malignant infections vs. ACs and patients with HAM/TSP (Gillet et al., 2011). In addition, the unique gene expression profile of PBMCs in HAM/TSP has manifested as disruptions in interferon signaling to the point that this pattern can be employed to distinguish HAM/TSP patients from ACs (Tattermusch et al., 2012) and ATLL patients (Fukutani et al., 2019).

All of these discoveries suggest that HAM/TSP is developed through a dynamic interaction between the host and the virus in all stages of the disease, starting from the proviral integration in active transcription sites (Gillet et al., 2011) and viral modification of host gene expression to cross-communication of infected cells with the immune system and negative selection of active clones to translocation of the virus to CNS. Despite these improvements, further research is required to fully understand the unknown aspects of the disease, which have been highlighted in this article.

## Author Contributions

SA performed systematic and data collection and was a major contributor in writing the manuscript. MT-R provided with the illustrations and was a minor contributor to editing the manuscript. GM was a minor contributor in data curation and in writing the manuscript. S-HM conceptualized the review article, had a minor contribution to writing, and provided oversight, critical evaluation, and verification of the manuscript. All authors read and approved the final manuscript.

## Conflict of Interest

The authors declare that the research was conducted in the absence of any commercial or financial relationships that could be construed as a potential conflict of interest.
